# Cytotoxic Evaluation of the New Composite Resin through an Artificial Pulp Chamber

**DOI:** 10.1155/2022/5100816

**Published:** 2022-11-16

**Authors:** Luca Marigo, Alessio Triestino, Raffaella Castagnola, Federica Vincenzoni, Massimo Cordaro, Enrico Di Stasio, Alvaro Mordente, Giuseppina Nocca

**Affiliations:** ^1^UOC Odontoiatria Generale e Ortodonzia, Dipartimento Scienze dell'Invecchiamento, Neurologiche, Ortopediche e della Testa-Collo, Fondazione Policlinico Universitario A. Gemelli, IRCCS, Largo Agostino Gemelli 8, Rome 00168, Italy; ^2^Dipartimento di Testa-Collo e Organi di Senso, Università Cattolica del Sacro Cuore, Largo Agostino Gemelli 8, Rome 00168, Italy; ^3^Dipartimento di Scienze Biotecnologiche di Base, Cliniche Intensivologiche e Perioperatorie, Sezione di Biochimica, Università Cattolica del Sacro Cuore, Largo F. Vito, 1, 00168 Rome, Italy; ^4^Fondazione Policlinico Universitario A. Gemelli, IRCCS, Largo A. Gemelli 8, 00168 Rome, Italy

## Abstract

The aim of this study was to analyse the cytocompatibility of Surefil One (SuO) with respect to the release of monomers from the material. The following reference materials were chosen: SDR Flow Plus (SDR, Dentsply Sirona, Konstanz, Germany), One Q Bond (Q, Dentalica, Milan, Italy), and Ketac (K, 3M-ESPE, USA). Fifteen dentin discs (2 mm thickness and diameter) were obtained from 15 third molars and were used in this study. After dentin disc permeability measurement, murine fibroblasts were grown, and the pulp surface of the dentinal disc was placed in direct contact with the cells immersed in DMEM. The experimental materials were positioned on the occlusal side of each dentinal disc until a uniform thickness of 2 mm was obtained. Then, the discs were inserted into an artificial pulp chamber for 24 hours to assess the cytocompatibility. Afterwards, the moles of monomers leached from the specimens in DMEM were determined using HPLC. Statistical analysis was performed using ANOVA (*p* < 0.05). Under the experimental conditions, the toxic effect induced by all tested materials was slight or absent. Diurethane dimethacrylate and acrylic acid were not found in the culture media. It is concluded that all materials have good cytocompatibility consistent with the nondeterminability of the monomers released after polymerization.

## 1. Introduction

Composite resins were introduced on the market more than 60 years ago and have undergone progressive improvements. Among these is the production of bulk-fill composites and self-adhesive composite resins to simplify the reconstructive procedures and operator-employee processes to minimize any operational error.

The rationale associated with the use of bulk-fill composite resins is to simplify the reconstructive procedures. However, the inclusion of composite materials in a single increment has been contraindicated for a long time for two main reasons [1]: conventional materials need to be positioned in increments of no more than 2 mm to ensure proper conversion of monomers on the cavity bottom, especially class I and class V cavities, characterized by a high cavity configuration factor (C-factor), and the application of composite resins in a single increment can cause excessive stress.

To overcome these limitations and consequently allow the application of these materials in single increments, some improvements were introduced over time [1]. Materials with reduced filler content and, in any case, fillers able to permit the transmission of light in depth, with more efficient initiators and with minor stress during the polymerization phase, were produced.

The creation of a correct adhesive interface, which is essential for applying traditional composite resins, represents a daily challenge for clinicians due to the hydrophobicity of these materials. The need to simplify adhesive procedures has led researchers to develop universal adhesive agents, i.e., containing etchants, primers, and adhesives within a single product, to further reduce the clinical steps and, consequently, the margins of error [2].

Flow self-adhesive composite resins have recently been introduced to the market to combine the advantages of universal adhesive systems and flowable composite resins. The clinical advantages of these materials include [2] ease of use because they do not require the separate application of etching, primer, and adhesive, prevention of procedural errors associated with the clinical application of conventional adhesive agents, such as overdrying or excessive moisture of the dentinal substrate; and reduction of chairside operating times.

Based on these undisputed advantages, many doubts remain regarding the duration and clinical efficacy of these materials because studies on their mechanical, physical, and biological properties are also scarce [3].

A careful analysis of research in conservative dentistry has highlighted the growing demand for simplicity in the field of reconstructive procedures. To solve these problems, a new self-adhesive bulk-fill composite, Surefil One (SuO, Dentsply Sirona; Konstanz, Germany), was recently introduced on the market [4].

The innovation of this material consists of a modified polyacid capable of combining the self-adhesive properties of glass ionomer cement with the ability of the functional groups of the resinous monomers to create cross-links. The modified polyacid has one functional group capable of binding to the calcium ions of enamel and dentin and another functional group capable of cross-linking and polymerizing with other compounds, such as inorganic filler, resin matrix, and polyacids [4].

The presence of a dual activator allows the material to be applied as a bulk-fill composite.

The use of polyacids in the formula of the material requires the presence of water due to the insolubility of polyacids in traditional composite resins. Each component of the material must be compatible with water and stable in an aqueous environment. Consequently, the classic reactive diluents must be replaced with water-soluble and hydrolytically stable molecules [4].

Yao et al., in a recent *in vitro* study, argue that the interaction between this material and the dental substrate should occur through the acid functional groups of the monomeric and polymeric components. These should not only ensure microretention by etching the surface of the substrate but also create ionic bonds with the calcium present in hydroxyapatite. The authors, however, support the urgency of new *in vitro* studies to elucidate the mechanisms underlying self-adhesion [5]. However, the exploration of the mechanical properties of bulk-fill materials is not the only important research field; many papers have evaluated the cytotoxicity induced by these materials [6–11]. Unfortunately, due to the different evaluation approaches, the results are—in some cases—contradictory. However, in most of the works, the results obtained seem to indicate that the cytotoxic effect caused by bulk-fill materials is comparable to that of traditional ones [12].

Therefore, the aim of this pilot study was to help settle this issue, evaluating the toxicity induced by a new-generation bulk-fill material (SuO) and relating it to the release of monomers. As reference materials, other bulk-fill materials, such as SDR Flow Plus (SDR, Dentsply Sirona, Konstanz, Germany), One Q Bond (Q, Dentalica, Milan, Italy), and one conventional glass ionomer material, Ketac (K, 3M-ESPE, USA), were chosen. The composition of the tested materials is reported in Table 1.

The null hypothesis to be tested was that there are no differences in cytotoxic effects induced by SuO with respect to other materials. Moreover, because the permeability of dentin is decisive in the cytotoxic effect of these materials, an artificial pulp chamber (APC), similar to the one developed by Outhwaite et al. [13], was used. This device is still utilized, albeit with appropriate modifications [14–16]. The use of models with a dentin barrier to study the cytocompatibility of material represents a great evolution since it allows us to obtain a greater similarity with the characteristics observed *in vivo* [17, 18]. Consequently, the use of an APC represents an effective experimental model for evaluating the cytotoxicity of numerous materials with different application techniques [19].

## 2. Materials and Methods

### 2.1. Chemicals and Reagents

Cell culture medium and reagents, ethanol (EtOH and HPLC grade), and acetonitrile (CH_3_CN, HPLC grade) were purchased from Sigma-Aldrich (Milan, Italy). Methyl alcohol (CH_3_OH, HPLC grade, Prolabo, France) and ultrapure water (obtained by a P. Nix Power System apparatus, Human, Seoul, Korea) were used for HPLC analyses.

### 2.2. Dentin Disc Preparation

Fifteen mandibular and maxillary human third molars (numbered from 1 to 15) without fractures, defects, or morphological alterations were used for this study. The inclusion criteria included teeth with complete root formation. The authorization for the use of the biological material was obtained from each patient. After removing the surface debris and periodontal ligament remnants, the teeth were preserved in 0.1% thymol solution at 4°C for 48 h, according to da Fonseca Roberti Garcia et al. [20]. The roots of the tooth elements were dissected 2 mm from the cement-enamel junction using a precision cutting machine with a water-cooled diamond saw (Isomet 1000, Buehler LTDA, USA). Transverse cuts were made until a flat dentinal surface was reached immediately above the region of the pulp horns. For each tooth, dentin discs were made in the region of the middle third of the crown with a thickness of 2.2 mm. These discs were sectioned, keeping the section plane as close as possible to the pulp region and avoiding the inclusion of projections of the pulp horns. The obtained sections were analysed by an optical microscope (Olympus SZX7, Italy) to confirm the absence of enamel on the occlusal surface and defects resulting from the projections of the pulp horns on the pulp surface. The surfaces of the dentine discs were finished and smoothed using sheets of abrasive paper in order of decreasing grain size (# 600 and # 400, United Abrasives, USA) until reaching a dentinal thickness of 2 mm, measured by a digital calliper (Digital ABS calliper 0-450 mm, Mitutoyo, Japan) [14, 20] with a precision margin of 0.01 mm.

Subsequently, the total area of the dentinal discs was reduced by means of a cylindrical diamond bur (Komet 835KR, Germany) mounted on a high-speed handpiece (Kavo EXPERTmatic, Germany) to obtain discs with a final diameter of 2 mm.

Dentin discs were stored in a phosphate buffer solution (PBS), pH 7.2, at 4°C until the dentin permeability was measured [14].

### 2.3. Artificial Pulp Chamber (APC)

**Figure 1:**
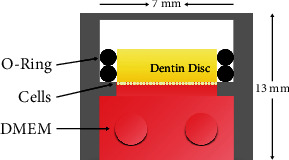
The APC with two silicone “O-Ring,” the dentin discs, the cells, and the DMEM.

In APC, the dentin discs were placed inside a cylindrical structure of polyurethane obtained by a 3D printer (Dremel DigiLab, Nederland). The lateral surfaces of the dentin discs were separated from the walls of the APC by two silicone “O-RING.” The pulp surface of the dentin discs was put in contact with the culture medium. The materials under examination were placed on the occlusal surface of the dentin disc (Figure 1).

### 2.4. Hydraulic Conductance Measurement

The hydraulic conductance (Ci) was calculated by filtration to measure the dentinal permeability. This value is determined by applying constant hydrostatic pressure to the dentinal disc for a period and using the following equation:

(1) $$\begin{array}{c} \text{Ci}=\frac{\text{Jv}}{A∙∆P∙T}, \end{array}$$

where Ci is the hydraulic conductance expressed in *μ*L cm^−2^ min^−1^cmH_2_O^−1^; Jv is the volume of the fluid expressed in *μ*L, i.e., the distance travelled by a microbubble of air, expressed in mm; *A* is the area of the dentinal surface expressed in cm^2^; Δ*P* is the pressure gradient expressed in cm H_2_O; and *T* is the time required for fluid movement expressed in minutes.

Each dentinal disc was placed in the APC to determine the hydraulic conductance.

Both surfaces of the dentin discs were treated with 17% ethylenediaminetetraacetic acid (EDTA) to eliminate the smear produced during the previous phases and copiously treated with rinses of distilled water. A polyvinyl chloride tube, with an internal diameter of 9 mm, attached to the central compartment of the APC was connected to a column of water of 21.5 cm. The disc remained under this pressure for 5 minutes. After this period, the movement of a microbubble present in the cannula was measured for 5 minutes, and the values obtained (Jv) were transformed into dentinal conductance values using the equation previously described [14].

Hydraulic conductance values were analysed using ANOVA.

Subsequently, each dentinal disc and APC were sterilized using UV rays and a 40 W lamp for thirty minutes.

The discs were placed in sterile PBS, pH 7.2, at 4°C until the cytotoxicity analysis.

### 2.5. Cell Culture Conditions

Murine fibroblast 3T3-Swiss (Istituto Zooprofilattico, Brescia, Italy) was grown in a 5% CO_2_ atmosphere at 37°C in DMEM (Dulbecco modified Eagle's medium) with HEPES (10 mM), glucose (4.5 g/L), NaHCO_3_ (3.7 g/L), penicillin (100 units/mL), streptomycin (100 g/mL), and 10% foetal calf serum.

### 2.6. Cytocompatibility Assays

The cytocompatibility of the APC device was evaluated by eluate and direct mode utilizing the 3-(4,5-dimethylthiazol-2-yl)-2,5-diphenyltetrazolium bromide (MTT) test, according to a protocol previously described [21].

In the eluate, the APC device (previously sterilized) was immersed in 20 mL of DMEM and incubated for 15 days at 37°C. Thus, the 3T3-Swiss fibroblasts were seeded in 96-well plates at a density of 10,000 cells/well in 0.2 mL of DMEM. After 24 h, the culture medium was replaced with a different amount of eluate (0.2 mL, 0.15 mL, or 0.10 mL). The final volume was made up to 0.20 mL with fresh medium. The cells with 0.2 mL of DMEM were used as controls. After 24 hours of incubation, cell viability was evaluated [21]. Briefly, 20 *μ*L of a solution of MTT in PBS (phosphate buffer, 5 mg mL^−1^) was added to the medium (0.20 mL). After incubation for 4 h at 37°C, the produced formazan crystals were solubilized with a solution of HCl in isopropanol (4 × 10^−2^ M, 0.20 mL). The absorbance (abs) of the solutions in each well was determined using an automatic microplate photometer (ELx800; BioTek, Bad Friedrichshall, Germany) at a wavelength of 562 nm. Each experiment was performed in sextuplicate and repeated four times. The cytotoxicity was calculated according to the following equation: [(abs control − abs sample)/abs control] × 100.

For direct toxicity tests, the cells were seeded in 24-well plates at a concentration of 20,000 cells/well in 2 mL of DMEM. After 24 hours, a sterilized device was added to the cells and left in contact for 48 hours. The MTT assay was then performed. The experiment was repeated 2 times.

**Figure 2:**
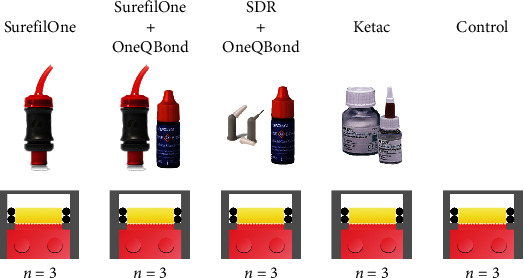
The four experimental groups and the control group. *n*: number of specimens.

After ascertaining the complete cytocompatibility of the APC device, the experiment with materials was carried out with the following groups (Figure 2):

- “SuO” group: “Surefil One” material (Dentsply Sirona, Konstanz, Germany) applied in direct contact with dentin
- “SDR+Bond” group: “SDR Flow Plus” material (Dentsply Sirona, Konstanz, Germany) applied after carrying out the adhesive procedures by acid etching with 37% orthophosphoric acid “Axia Etch Jumbo” (Dentalica, Milan, Italy) and application of one-component etch-and-rinse One Q Bond CGT adhesive (Dentalica, Milan, Italy)
- SuO+Bond group: “Surefil One” material (Dentsply Sirona, Konstanz, Germany) applied after carrying out the adhesive procedures by acid etching with 37% orthophosphoric acid “Axia Etch Jumbo” (Dentalica, Milan, Italy) and application of one-component etch-and-rinse One Q Bond CGT adhesive (Dentalica, Milan, Italy)
- Ketac group (K): “Ketac” material (3M-ESPE, USA) was applied after mixing the powder and liquid components in a 3 : 1 powder-liquid ratio
- Control group (C): no material was applied on the occlusal side of the dentin
- Each group consisted of 3 dentine discs, each of which was contained within the respective APC

Each APC was then placed inside a 24-well plate, with the occlusal side of the dentinal surface towards the bottom of the plate. A total of 3 × 10^4^ cells in a volume of 20 *μ*L were placed on the dentin disc in contact with the pulp side of the dentinal surface. The cells were kept in this position in an incubator at 37°C and 5% CO_2_ for 30 minutes to allow the initial cell adhesion on the pulp side of the dentinal surface. After this period, 1 mL of DMEM was added to each APC. The 24-well plates containing the APCs were then placed in an incubator at 37°C and 5% CO_2_ for an additional period of 48 hours to allow the cells to proliferate on the dentin substrate [22]. At the end of the 48-hour incubation period, the culture medium was replaced by 1 mL of DMEM without foetal bovine serum. Each APC was placed with the occlusal surface of the dentine disc facing up. In this way, while the pulp surface of the dentinal disc was placed in direct contact with the cells immersed in DMEM, the occlusal surface remained to allow the application of the materials under study [23].

#### 2.6.1. Application of the Tested Materials

Surefil One restorative material was applied following the manufacturer's instructions. The product was positioned on the occlusal side of each dentinal disc until a uniform thickness of 2 mm was obtained, and then, the Starlight Pro (Mectron, Italy) curing light was applied with an intensity of 1200 mW/cm^2^ for 20 seconds.

The glass ionomer cement “Ketac Universal Aplicap” (3M-ESPE) was applied using a microbrush after mixing the powder and liquid components in a 3 : 1 ratio proposed by the manufacturer. This material was applied directly on the occlusal side of the dentinal disc, obtaining a thickness of 2 mm (3M-ESPE).

All adhesive procedures were performed under sterile conditions. After proceeding with the application of the materials, for each group under consideration, each APC was placed in an incubator at 37°C and CO_2_ at 5% for 24 hours.

After the 24-hour incubation, the MTT test was performed.

The dentinal discs were removed from their respective APCs and placed in a new 24-well plate with the pulp side containing the cells positioned upwards. Then, 900 *μ*L of DMEM and 100 *μ*L of MTT solution were added to each well. The cells were subsequently incubated at 37°C for 3 hours. The DMEM was removed and stored at -20°C for subsequent chromatographic analysis.

Thus, the culture medium containing the MTT solution was replaced with 400 *μ*L of acidified isopropanol solution in each well.

Three aliquots (100 *μ*L) from each well were transferred to a 96-well plate. The produced formazan was evaluated as previously reported.

Specimens were rated as slightly, moderately, or severely cytotoxic when the toxic effects, relative to controls, were <30%, between 30% and 60%, or >60%, respectively [11, 24].

### 2.7. Monomer Leaching Evaluation

High-performance liquid chromatography (HPLC) was used to determine the moles of monomers (acrylic acid (AA) and diurethane dimethacrylate (DUDMA)) leached from specimens in DMEM. Three specimens (*n* = 3) were used for each material.

Before the HPLC analysis, the cell culture media were centrifuged (13000 g, 15 min) and filtered (0.45 *μ*m syringe filter, Whatman, Maidstone, Kent, UK). Finally, samples were diluted in acetonitrile (1 : 10) and analysed using a Thermo Finnigan HPLC equipped with two pumps, a diode array detector and an autosampler.

The assays (50 *μ*L injected volume) were performed at a wavelength of 214 nm with a C-18 (5 *μ*m) Supelco reversed-phase column (250 × 4.6 mm) using a mixture of water (A) and acetonitrile (B) gradient from 40% to 20% A (30 min) (for DUDMA) as the mobile phase (0.7 mL/min) [11].

For AA, we used, for the first time, the following HPLC conditions: flux 0.8 mL/min, and the mobile phase was composed of water (A) and methanol (B) gradient from 75% to 66% of A (in 7 min) and from 66% A to 75% An in 1 min. The assays (50 *μ*L injected volume) were performed at a wavelength of 240 nm with a C-18 (5 *μ*m) Supelco reversed-phase column (250 × 4.6 mm).

Before and after each analysis, a calibration line was performed with standard solutions of AA and DUDMA.

### 2.8. Statistical Analysis

Each value represents the mean of a different number of experiments. All results are expressed as the mean ± standard deviation (SD). The group means were compared by analysis of variance (ANOVA) followed by multiple comparisons of means by Student-Newman-Keuls. If necessary, a comparison of means by Student's *t*-test was used: *p* < 0.05 was considered significant.

## 3. Results

### 3.1. Hydraulic Conductance

The hydraulic conductance values, calculated using Equation (1) [14, 22], are shown in Table 2. No statistically significant differences (*p* > 0.05) were present between the hydraulic conductance values in the 5 groups.

### 3.2. Cytotoxicity

**Figure 3:**
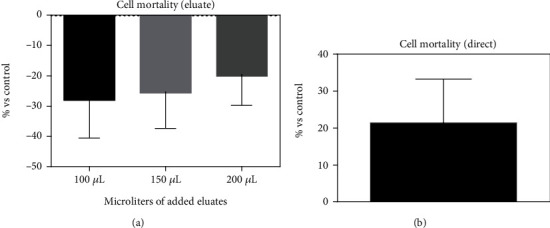
Cytotoxic effects of APC on 3T3-Swiss fibroblasts. Each experiment was repeated four times.

APC induced no toxic effects in the eluate modality (Figure 3(a)). Furthermore, the statistical analysis between the abs means calculated in the samples of the three groups with different concentrations of eluate (100 *μ*L, 150 *μ*L, and 200 *μ*L) did not reveal any statistically significant difference (*p* > 0.05). The APC-induced toxicity assessed in direct mode was slight, as shown in Figure 3(b).

#### 3.2.1. Cytotoxicity Induced by Tested Materials in APCs

**Figure 4:**
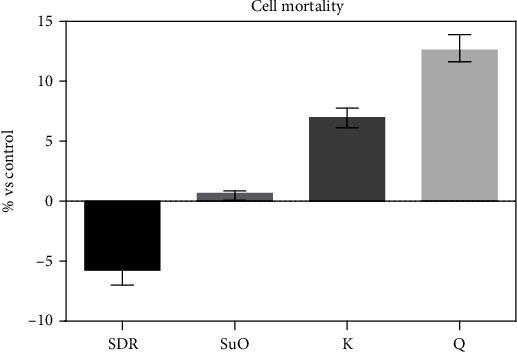
Cytotoxic effects of materials—added through APC—on 3T3-Swiss fibroblasts.

Under the experimental conditions used, the toxic effect induced by all tested materials was slight (1-12%) or absent (Figure 4). No significant differences were observed when analysing the cytotoxic effects induced by the different materials (Figure 4). The effect size (size d) calculated on the basis of the experimental absorbance data was 0.29 (small effect), thus confirming a very small difference among subgroups in our pilot study, independent of the number of measures. Therefore, the calculated power on the comparison between the already acquired 3 repeated measures for each group is 19%. Finally, on the basis of our data, an unachievable sample size of 200 experiments should have been performed with a power of 80% and *a* = 0.05.

### 3.3. Monomer Leachability

**Figure 5:**
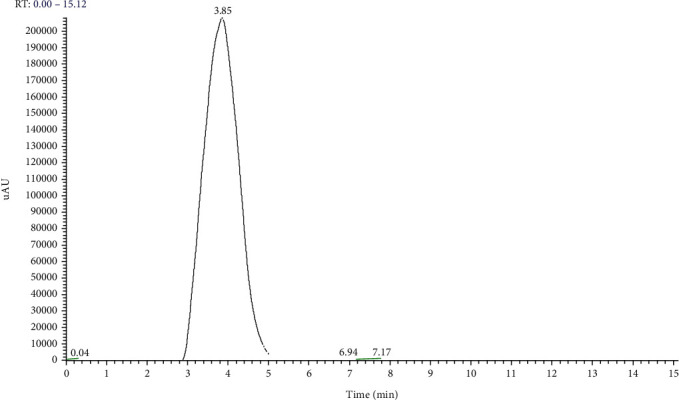
Chromatographic profile of pure DUDMA: this monomer was not detected in eluates derived from the materials.

The calibration lines performed for DUDMA highlighted the correctness of the chromatographic conditions used, and the retention time (Rt) for DUDMA was 3.85 min (Figure 5).

**Figure 6:**
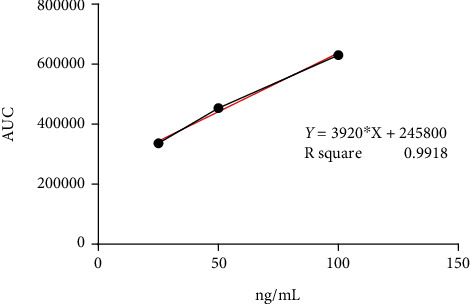
AA calibration line and regression curve. Concentration range 25.0–100 ng/mL.

**Figure 7:**
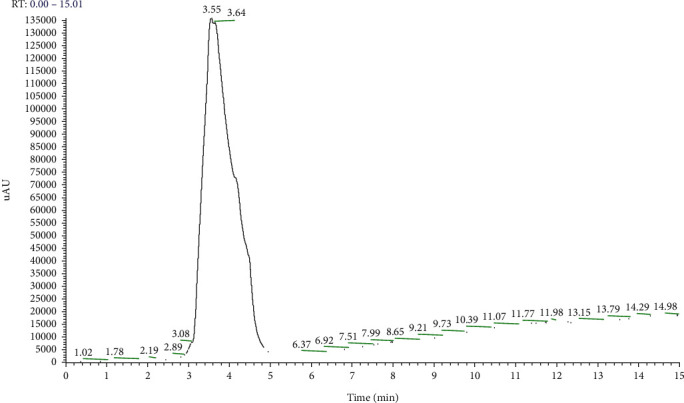
Chromatographic profile of pure AA: the monomer was not detected in eluates derived from the materials.

As the chromatographic method used for acrylic acid was unpublished yet, to be sure to detect the exact AA concentration in eluates, the method was tested for linearity using calibrating standard solution mixtures of AA in the concentration range of 25.0–100 ng/mL. Each determination was repeated two times, and each calibration curve was generated 5 times (*n* = 5). The obtained regression equation was *Y* = 3920^∗^*X* + 245800 with *r*^2^ = 0.9918, confirming the response linearity under the conditions used (Figure 6). Linearity was lost below a concentration of 25 ng/mL. The *r*^2^ value was equal to 0.9378 when the concentration range was 5 to 100 ng/mL. The Rt for AA was 3.5 min (Figure 7).

## 4. Discussion

The cytotoxicity induced by the materials analysed in this study was, for all, very slight. In a previous study, Şişman et al. [25] observed that the SDR bulk-fill composite showed the lowest cell viability compared to the control group. However, as already reported, these contradictory results are due to different analytical procedures; in fact, Şişman et al. used a “direct cell contact test” wherein the dental material and cells were placed in direct contact, different from the APC of this study, which is an “indirect contact test” [25]. Similarly, Attik et al. reported a slight cytotoxic effect for SDR [26]. Moreover, many studies have already shown the low cytotoxicity of Ketac and glass ionomer cement in general [27–29].

The results obtained in our study are of particular interest, especially considering the methodology applied: the adoption of a dentine barrier simulates the oral environment more closely *in vivo*, an option not available with other methods of analysis. In fact, this method is also indicated as preferable by the ISO 7405 standard [30]. Obviously, in preparation for the use of the dentin barrier inside the APC, it is important to verify that the hydraulic conductance of the dentine is the same for all samples; otherwise, there would be differences in the concentration of monomers able to reach the cells. The results obtained in our study showed that the permeability of the different samples was not significantly different; thus, our results are not dependent on this parameter. However, it is not possible to translate the results obtained *in vitro* into a clinical situation even if the APC represents a good device to evaluate cytocompatibility. Moreover, this *in vitro* model, like any other appropriate model, helps to reduce the number of tests to be performed *in vivo*.

Another observation derived from our results is that the slight cytotoxic effect observed is perfectly consistent with the low release of methacrylic monomers. Because the HPLC technique is considered the most suitable method to evaluate the type and concentration of monomers released from composite resins [31], in this study, HPLC was used to detect AA and DUDMA release. AA is a component of SuO and, in a minimal percentage, of Ketac, while DUDMA is present in SDR. Moreover, SuO combined the properties of the glass ionomer with those of the composite. Common chemical components and properties led us to use Ketac as a control.

However, we did not detect any monomers in our samples.

Other studies have evaluated the release of monomers from SDR [31–33]: the authors found a very small concentration of DUDMA in the samples. The differences with respect to our results are due to the different *in vitro* techniques used. In fact, none of the above-reported papers used indirect contact between cells and materials, and experiments were performed by immersing the samples directly in media without using APC [32].

Even if the presence of these monomers in the culture media of our samples has not been determined, it does not mean that no release has occurred, simply that regardless of the amount released, it is below the dose able to induce 50% cell mortality (TC_50_), and it is below the sensitivity threshold of our measurement system, despite the high sensitivity and reproducibility of the chromatographic technique used. Moreover, the chromatographic procedure developed in this study to quantize acrylic acid represents, in terms of simplicity and speed of execution, notable support in the evaluation of this monomer present in many composite resins compared to other protocols. [34, 35].

The certainty of having good cytocompatibility characteristics always constitutes a reassurance for the clinician that he will have to use a material for the restoration. However, there are many situations that affect the longevity of a restoration placed in the oral cavity. Thus, *in vivo* studies are necessary to evaluate a biomaterial.

There are still few studies regarding the use and real effectiveness of Surefil One. An annual clinical study by Rathke et al. of 60 reconstructions in 41 patients reported encouraging data [36]. The reconstructions were evaluated at 3 months and 1 year. The annual failure rate is 2%, comparable to traditional composite resin restorations [37].

## 5. Conclusion

Based on the perfect correlation between low toxicity and low monomer release and thanks to the device used to test the toxicity, it can be concluded that the results of this pilot study can help to clarify the plethora of different results regarding the toxicity caused by bulk-fill materials.

Moreover, because the toxicity induced by all materials was slight, we can conclude that our null hypothesis was correct.

However, as the composition is different from traditional resins, it will be necessary to perform more clinical studies on SuO to understand its real potential and its long-term performance.

## Figures and Tables

**Table 1:** Analysed materials and their composition.

SDR flow, Dentsply Sirona	Ketac, 3M-ESPE	Surefil One, Dentsply Sirona	One Q Bond, Dentalica
(i) Ba-Al-fluorosilicate glass (ii) St-Al-fluorosilicate glass (iii) Modified diurethane dimethacrylate resin (iv) Modified bisphenol A ethoxylated (v) Triethylene glycol dimethacrylate	Powder: (i) Glass powders (ii) Polycarboxylic acid	(i) Al-P-Sr-Na-fluorosilicate glass (ii) Highly dispersed silicon dioxide (iii) Water (iv) Acrylic acid (v) Polycarboxylic acid (vi) Bifunctional acrylate (vii) Self-polymerizing initiator (viii) Camphorquinone	(i) Ethanol (ii) Acetone (iii) Hydroxyethyl methacrylate (iv) Hydroxyethyl dimethacrylate (v) Methylene-ethylidene-bis-fenilenossido-etandiile-methacrylate (vi) Urethane dimethacrylate (vii) Camphorquinone
	Liquid: (i) Water (ii) Tartaric acid (iii) Copolymer of maleic acid and acrylic acid		

**Table 2:** Hydraulic conductance values of dentin, calculated on fifteen samples within five groups.

Group	Dentin specimen	Bubble shift	∆*P* (cm H_2_O)	Area (cm^2^)	*t* (min)	Hydraulic conductance (*μ*L cm^−2^ min-1 cm H_2_O^−1^)
SuO	14	0.037	21.43	0.1256	5	0.00275
	6	0.074	21.43	0.1256	5	0.00550
	3	0.037	21.43	0.1256	5	0.00275
SDR+Bond	8	0.037	21.43	0.1256	5	0.00275
	2	0.074	21.43	0.1256	5	0.00550
	5	0.037	21.43	0.1256	5	0.00275
SuO+Bond	9	0.074	21.43	0.1256	5	0.00550
	15	0.037	21.43	0.1256	5	0.00275
	1	0.074	21.43	0.1256	5	0.00550
Ketac	7	0.074	21.43	0.1256	5	0.00550
	13	0.074	21.43	0.1256	5	0.00550
	11	0.037	21.43	0.1256	5	0.00275
Control	4	0.037	21.43	0.1256	5	0.00275
	12	0.037	21.43	0.1256	5	0.00275
	10	0.074	21.43	0.1256	5	0.00550

## Data Availability

All research data are available in the manuscript.
